# Genome-Wide Association Study of Body Weight and Body Size Traits in Wenshang Barred Chickens at the Late Rearing Stage

**DOI:** 10.3390/ani16203255

**Published:** 2026-10-16

**Authors:** Zhongsheng Chen, Jinxing Zhou, Yan Sun, Wei Liu, Jie Liu, Jie Wang, Haixia Han, Dan Hao, Xinlong Zhu, Qiuxia Lei, Yan Zhou, Dingguo Cao, Fuwei Li, Chuanyan Che

**Affiliations:** 1 College of Animal Science, Anhui Science and Technology University, Chuzhou 233100, China; chenzhongsheng0911@163.com (Z.C.); zhoujx@ahstu.edu.cn (J.Z.); 2 Poultry Institute, Shandong Academy of Agricultural Sciences, Jinan 250100, China; sunyan881102@163.com (Y.S.); lwteam@126.com (W.L.); jas_saas@163.com (J.L.); wj_saas@163.com (J.W.); hanhaixia@163.com (H.H.); haodan111121@163.com (D.H.); zhuxinlong623@163.com (X.Z.); lei_qiuxia@163.com (Q.L.); sally7919@163.com (Y.Z.); cdgjqs@163.com (D.C.); 3 Anhui Province Key Laboratory of Animal Nutritional Regulation and Health, Chuzhou 233100, China; 4 Jinan Key Laboratory of Poultry Germplasm Resources Innovation and Healthy Breeding, Jinan 250100, China; 5 Shandong Provincial Key Laboratory of Livestock and Poultry Breeding, Jinan 250100, China

**Keywords:** Wenshang Barred chicken, GWAS, body weight, body size, whole-genome resequencing

## Abstract

The late rearing stage is a critical period for the development of body weight and body size traits in chickens. In this study, we investigated the genetic architecture underlying these traits in 925 healthy 16-week-old Wenshang Barred chickens, with the aim of identifying candidate genes and genetic markers for trait improvement. We performed a genome-wide association study, linkage disequilibrium analysis, genotype–phenotype effect analysis, and enrichment analysis for body weight and seven body size traits. Heritability estimates for these traits ranged from 0.18 to 0.48. The study identified 89 candidate genes, including *SETDB2*, *CAB39L*, *RB1*, *TRIM13*, *LDB2*, and *LCORL*. In addition to the classic growth-associated region at GGA4:72.48–76.42 Mb, we identified an important region at GGA4:68.24–70.22 Mb, located approximately 2.3 Mb upstream, as well as an important region at GGA1:168.03–170.24 Mb. Functional enrichment analysis revealed that candidate genes were nominally enriched (*p* < 0.05) in amino sugar and nucleotide sugar metabolism, biosynthesis of nucleotide sugars, calcium signaling, and neuroactive ligand–receptor interaction pathways. Collectively, our findings clarify the genetic basis of body weight and body size traits during the late rearing stage and may provide a basis for further evaluation of genetic markers in indigenous chicken breeding programs.

## Simple Summary

Body weight and body size traits are critical indicators in poultry production, as they directly reflect skeletal development and muscle growth. However, indigenous chicken breeds typically exhibit slow growth during the late rearing period, the phase prior to marketing or the onset of lay, which substantially increases production costs. The genetic regulatory mechanisms of these traits remain poorly understood, limiting the effectiveness of targeted breeding programs for high-quality indigenous chicken breeds. Given that the growth traits during this stage are closely associated with slaughter and laying performance, improving them could enhance both production efficiency and economic returns. In this study, we conducted a genome-wide association study using 925 healthy 16-week-old Wenshang Barred chickens of both sexes to identify genetic markers and candidate genes associated with body weight and body size traits. Heritability estimates for these traits ranged from 0.18 to 0.48. The study revealed 4080 genome-wide significant single-nucleotide polymorphisms, 89 candidate genes, three important candidate regions, and four candidate pathways. Collectively, these findings provide a comprehensive genetic characterization of body weight and body size traits in Wenshang Barred chickens during the late rearing stage and may provide a basis for further evaluation of genetic markers in indigenous chicken breeding.

## 1. Introduction

The Wenshang Barred chicken originates from Wenshang County, Shandong Province, China, and is a renowned indigenous dual-purpose breed valued for both eggs and meat. It is characterized by strong adaptability to adverse conditions, excellent egg-laying performance, flavorful meat, and attractive plumage. The breed is currently included in China’s National Livestock and Poultry Genetic Resources Conservation List and registered as a national geographical indication product; it has unique genetic and cultural value and holds an important position in China’s specialty poultry industry. Body weight and body size traits are important indicators for evaluating poultry production characteristics, as they directly reflect skeletal development and muscle growth. Consequently, these growth-related traits are widely employed as key selection criteria in breeding programs aimed at improving production efficiency and economic returns [1,2].

Genome-wide association study (GWAS) can identify associations between genetic variation markers across the whole genome and phenotypic traits [3,4]. In the field of poultry genetics and breeding, GWAS has been widely used to elucidate the genetic basis of multiple important economic traits in chickens, including carcass performance [5], egg production performance [6], feed efficiency [7,8], and secondary sexual characteristics [9]. Meanwhile, this technique has successfully identified key genes associated with growth traits, such as *LDB2*, *NCAPG*, *LCORL*, *KCNIP4*, and *SLIT2* [10,11].

Indigenous chicken breeds typically exhibit slower growth rates compared to their commercial counterparts. Generally, skeletal and muscle growth predominate before 8 weeks of age, with growth rate peaking at approximately 10 weeks of age, and the growth rate is relatively slow during the late rearing stage, which seriously increases production costs [12,13,14]. The late rearing stage is a critical stage before market age and the onset of egg laying, and growth traits during this period are closely related to carcass traits and egg production performance. Strengthening selection for growth traits in chickens during this period can significantly improve production performance and economic benefits [15,16,17,18].

However, the genetic regulatory mechanisms underlying body weight and body size traits in indigenous chicken breeds at the late rearing stage remain poorly understood. Systematic genome-wide studies are still scarce, which seriously restricts the progress of targeted breeding of high-quality indigenous chicken breeds. Therefore, in this study, 925 healthy Wenshang Barred chickens at the late rearing stage, including 597 females and 328 males, were selected as the study population. Using whole-genome resequencing, we obtained genome-wide single-nucleotide polymorphisms (SNPs) and applied a single-trait linear mixed model to conduct GWAS for body weight and seven body size traits, including chest breadth, chest depth, keel length, body oblique length, pelvic width, shank length, and shank circumference. The primary objectives were to identify significant genetic loci and candidate genes influencing these traits and to provide a preliminary basis for evaluating genetic markers for potential use in selection and conservation programs in indigenous chicken breeding.

## 2. Materials and Methods

### 2.1. Ethics Statement

All animal handling and experimental procedures involving chickens in this study were conducted in accordance with the guidelines of the Ministry of Science and Technology of the People’s Republic of China (Beijing, China). The study protocol was approved by the Institutional Animal Care and Use Committee of the Shandong Academy of Agricultural Sciences (SAAS; Jinan, China; approval No. SAAS-2024-G08; approved on 6 March 2024).

### 2.2. Resource Population and Phenotype Collection

A total of 925 healthy Wenshang Barred chickens, including 597 females and 328 males, were obtained from the Research Base for Healthy Layer Hen Production Engineering and Equipment of the Ministry of Agriculture and Rural Affairs (Dongying, China). From 1 day of age onward, all birds were reared and managed under uniform conditions in accordance with the Poultry Breeding Manual of the research base. At 16 weeks of age, body weight and body size traits were measured using an electronic scale, measuring tape, and vernier calipers, in accordance with the Chinese national agricultural industry standard Terminology and Measurement Methods for Poultry Production Performance (NY/T 823–2020). Phenotypic data were collected for body weight (BW), chest breadth (CB), chest depth (CD), keel length (KL), body oblique length (BOL), pelvic width (PW), shank length (SL), and shank circumference (SC).

### 2.3. Whole-Genome Resequencing

Blood samples were collected from the wing veins of all birds using sterile vacuum blood-collection needles and transferred into anticoagulant-containing vacuum tubes for whole-genome resequencing (Supplementary Table S1). Genomic DNA was extracted from whole-blood samples using the DNeasy Blood & Tissue Kit (QIAGEN, Hilden, Germany), according to the manufacturer’s instructions. DNA purity was assessed by measuring the absorbance ratio at 260 and 280 nm using a NanoDrop 2000 spectrophotometer (Thermo Scientific, Wilmington, DE, USA), and DNA concentration was accurately quantified using a Qubit fluorometer (Invitrogen, Carlsbad, CA, USA).

Genomic DNA that met the quality-control criteria was randomly fragmented to an average size of approximately 350 bp using a Covaris focused ultrasonicator (Covaris, Woburn, MA, USA). Sequencing libraries were constructed according to the manufacturer’s instructions using the TruSeq Library Construction Kit (Illumina, San Diego, CA, USA). Qualified libraries were sequenced on an Illumina NovaSeq 6000 platform (Illumina, San Diego, CA, USA) with 150-bp paired-end reads. Whole-genome resequencing was performed at an average depth of approximately 10× per individual, and the resulting reads were used for subsequent analyses.

### 2.4. Variant Calling, Imputation, and Quality Control

Raw sequencing reads were filtered using fastp (v0.20.0) according to the following criteria: (1) reads containing adapter sequences were removed; (2) reads in which the proportion of undetermined bases (N) exceeded 5% were removed; and (3) low-quality reads were removed when bases with a quality score of ≤10 accounted for more than 20% of the total read length.

The filtered paired-end sequencing reads were aligned to the chicken reference genome (GRCg7b, Ensembl release 107; URL: https://ftp.ensembl.org/pub/release-107/fasta/gallus_gallus/dna/, accessed on 6 April 2025) using the Burrows–Wheeler Aligner (BWA v0.7.13) with the parameters mem -t 4 -k 32 -M. The resulting BAM files were converted, sorted, and indexed using SAMtools (v1.3.1). Duplicate reads were subsequently removed using Picard (v1.119).

Following alignment, SNPs were identified for each sample using GATK HaplotypeCaller (v4.0.4.0). SNPs were filtered according to the following criteria: QD < 2.0, FS > 60.0, MQ < 40.0, SOR > 3.0, MQRankSum < −12.5, or ReadPosRankSum < −8.0. The resulting genotype data were then subjected to quality control using PLINK (v1.90b6.24). Only SNPs located on autosomes 1–28 were retained, and the following filtering criteria were applied: variant missingness (GENO) < 0.05, minor allele frequency (MAF) > 0.05, Hardy–Weinberg equilibrium test *p*-value (HWE) > 1 × 10^−6^, and individual sample missingness (MIND) < 0.20. This procedure generated a high-quality genotype dataset for subsequent analyses.

To improve the quality of genotype data in the study population, missing genotypes at variant sites already present in the genotype VCF file were imputed during phasing using Beagle (v5.4). Chromosomes 1–28 were processed separately using the chrom parameter, without specifying an external reference panel (ref), and the default conversion of 1 cM = 1 Mb was used. After imputation, the total genotyping rate was 100%, and the genotype concordance rate was 99.76%. The imputed genotype data were subsequently subjected to quality control using PLINK, with the following criteria: variant missingness (GENO) < 0.05, minor allele frequency (MAF) > 0.05, Hardy–Weinberg equilibrium test *p*-value (HWE) > 1 × 10^−6^, and individual sample missingness (MIND) < 0.05. Finally, 925 individuals and 7,235,426 SNPs were retained for subsequent analyses.

### 2.5. Genetic Parameter Estimation

Genetic parameters were estimated using Genome-wide Complex Trait Analysis (GCTA v1.93.3) software [19]. A genetic relationship matrix (GRM) was constructed based on the genotype data, and restricted maximum likelihood (REML) analysis was performed to estimate genetic parameters [20]. The linear mixed model was defined as follows:

*Y*_i_ = *X*_i_*b*_i_ + *Z*_i_*u*_i_ + *e*_i_

where *Y*_i_ is the vector of phenotypic observations for trait _i_; *X*_i_ and *Z*_i_ are the incidence matrices relating observations to the fixed-effect vector *b*_i_ and the additive genetic-effect vector *u*_i_, respectively; and *e*_i_ is the vector of random residual effects. The first eight principal components (PCs) derived from principal component analysis (PCA) and sex were included as covariates to correct for population stratification. The additive genetic-effect vector was assumed to follow *u_i_~N*(0, ${G\sigma }_{u}^{2}$), where *G* is the GRM among individuals and $\sigma_{u}^{2}$ is the additive genetic variance. Residual effects were assumed to follow *e_i_~N*(0, ${I\sigma }_{e}^{2}$), where *I* is an n × n identity matrix and *n* = 925 represents the number of individuals.

Heritability was estimated using a univariate restricted maximum likelihood (REML) model and calculated as follows:

$$h^{2}=\frac{\sigma_{u}^{2}}{\sigma_{u}^{2}{+\sigma }_{e}^{2}}$$

Genetic and phenotypic correlations were estimated using a bivariate REML model. The genetic correlation was calculated as follows:

$$r_{G}=\frac{\sigma_{u_{12}}}{\sqrt{\sigma_{u_{1}}^{2}\sigma_{u_{2}}^{2}}}$$

The phenotypic correlation was calculated as follows:

$$r_{P}=\frac{\sigma_{u_{12}}+\sigma_{e_{12}}}{\sqrt{{(\sigma }_{u_{1}}^{2}+\sigma_{e_{1}}^{2}{)(\sigma }_{u_{2}}^{2}+\sigma_{e_{2}}^{2})}}$$

In the above equations, *h*^2^ denotes heritability, *r_G_* denotes the genetic correlation, and *r_P_* denotes the phenotypic correlation. In the univariate model, $\sigma_{u}^{2}$ and $\sigma_{e}^{2}$ denote the additive genetic and residual variances, respectively. In the bivariate model, $\sigma_{u_{1}}^{2}$ and $\sigma_{u_{2}}^{2}$ denote the additive genetic variances of traits 1 and 2, respectively, while $\sigma_{e_{1}}^{2}$ and $\sigma_{e_{2}}^{2}$ denote their respective residual variances. The terms $\sigma_{u_{12}}$ and $\sigma_{e_{12}}$ denote the additive genetic covariance and residual covariance between the two traits, respectively.

### 2.6. Genome-Wide Association Study

To elucidate the genetic basis of BW and body size traits, a genome-wide association study (GWAS) was performed using a univariate linear mixed model (LMM) implemented in Genome-wide Efficient Mixed Model Association (GEMMA v0.98.5) software [21] on whole-genome sequencing data from chickens. The statistical model was formulated as:

*y* = *Wα* + *Xβ* + *u* + *e*

where *y* is the vector of phenotypic values; *W* is the matrix of covariates, including the intercept, sex, and PCs, which were included to control for population structure and potential confounding effects; *α* is the vector of corresponding fixed-effect estimates, including the intercept; *X* is the genotype vector for the tested genetic marker; *β* is the effect size of the tested genetic marker; *u* is the vector of random polygenic effects; and *e* is the vector of residual effects. The first eight PCs derived from PCA and sex were incorporated into the fixed-effect matrix *W* to correct for population stratification.

The Wald test was used to identify SNPs associated with BW and body size traits. Bonferroni correction was applied to control for multiple testing in the GWAS. Based on 7,235,426 SNPs evaluated across the genome, 0.05/7,235,426 ≈ 6.91 × 10^−9^ and 1/7,235,426 ≈ 1.38 × 10^−7^ were used as the genome-wide and suggestive significance thresholds, respectively. Manhattan and quantile–quantile (Q–Q) plots of the GWAS results were generated using the CMplot (v4.5.1) package in R (v4.6.0).

### 2.7. Gene Annotation

The positions and annotation information of SNPs significantly associated with the traits of interest in the GWAS were retrieved based on the chicken reference genome GRCg7b from Ensembl release 115 (URL: https://ftp.ensembl.org/pub/release-115/fasta/gallus_gallus/dna/, accessed on 15 September 2025). Significant SNPs were annotated using the Ensembl Variant Effect Predictor (VEP). Genes overlapping with, or located within 5 kb upstream or downstream of, significant SNPs were considered candidate genes.

### 2.8. Regional Association, Linkage Disequilibrium, and Genotype–Phenotype Effect Analyses

Regional association analyses were performed to further characterize the association signals surrounding significant SNPs. Linkage disequilibrium (LD) patterns within the identified significant association regions were examined using the IntAssoPlot (v0.99.21) package [22] in R. The genotype–phenotype effects of the lead SNPs within these regions were visualized using the ggplot2 (v4.0.3) package.

### 2.9. Functional Enrichment Analysis

To explore the biological processes and pathways potentially represented by the candidate genes, Gene Ontology (GO) and Kyoto Encyclopedia of Genes and Genomes (KEGG) enrichment analyses were conducted using the clusterProfiler (v4.20.0) package [23] in R. GO terms and KEGG pathways with a nominal *p* value < 0.05 were considered nominally enriched.

### 2.10. Statistical Analysis

Descriptive statistical analyses of the phenotypic data were performed using IBM SPSS Statistics 27.0 (IBM Corporation, Armonk, NY, USA). Genotype–phenotype effect analyses for significant SNPs were conducted in R.

Homogeneity of variances was assessed using Levene’s test. When the assumption of homogeneity of variances was met, one-way analysis of variance (ANOVA) followed by Tukey’s honestly significant difference (HSD) test was used for multiple comparisons. When the assumption was not met, the Kruskal–Wallis test followed by Dunn’s multiple-comparison test was applied.

## 3. Results

### 3.1. Phenotypic Characteristics of Body Weight and Body Size Traits at the Late Rearing Stage

Descriptive statistics for BW and body size traits measured at the late rearing stage (16 weeks of age) are presented in Table 1. A total of 925 individuals were evaluated. The Kolmogorov–Smirnov (K–S) test indicated that CB and PW were normally distributed, whereas BW, CD, KL, BOL, SL, and SC were approximately normally distributed. The mean BW was 1009.89 g, with an SD of 176.98 g and a CV of 17.52%. Among the body size traits, BOL had the highest mean value (190.98 mm), followed by KL (100.47 mm), SL (78.35 mm), CD (77.68 mm), PW (63.33 mm), CB (50.56 mm), and SC (33.56 mm).

The CVs ranged from 6.14% for PW to 17.52% for BW. Among the body size traits, CD showed the greatest phenotypic variability (CV = 14.25%), whereas PW showed the lowest variability (CV = 6.14%). The observed ranges were 558.00–1561.00 g for BW, 39.60–62.02 mm for CB, 50.82–110.00 mm for CD, 80.00–120.00 mm for KL, 150.00–230.00 mm for BOL, 51.82–74.01 mm for PW, 60.59–102.07 mm for SL, and 28.00–39.00 mm for SC. Collectively, the eight traits exhibited appreciable phenotypic variation in the study population.

### 3.2. Principal Component Analysis (PCA)

**Figure 1:**
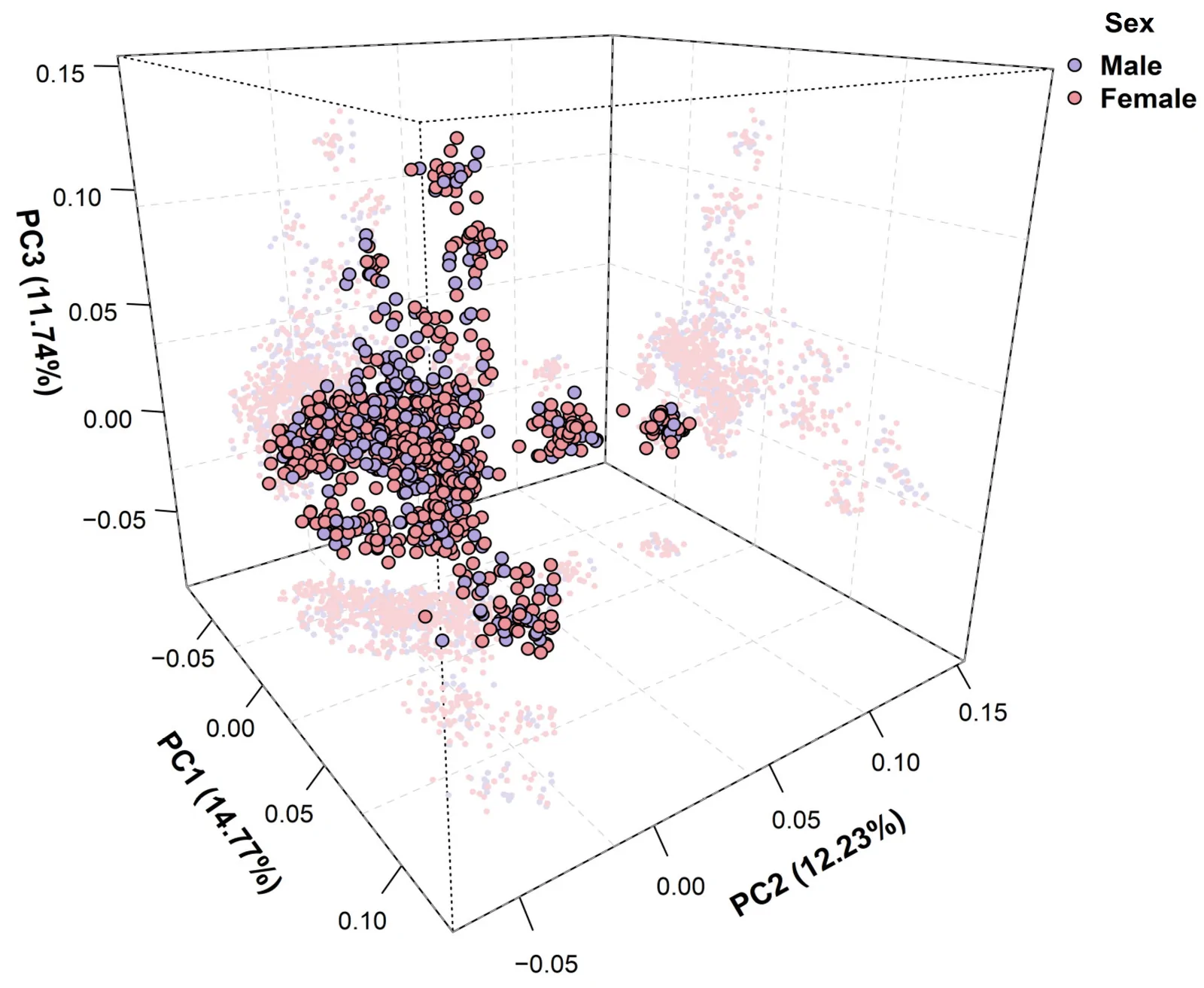
Three-dimensional principal component analysis of the study population. The analysis was based on genotype data from 925 Wenshang Barred chickens. Each point represents one chicken; purple and pink indicate male and female chickens, respectively. The first three principal components (PC1, PC2, and PC3) explained 14.77%, 12.23%, and 11.74% of the variation, respectively.

Principal component analysis (PCA) based on the genotype data of the study population is shown in Figure 1, and the three-dimensional PCA plot provides a clearer visualization of population structure. In the PCA plot, individuals showed a roughly cone-shaped distribution, suggesting variation in genetic structure within the study population. Most individuals are concentrated in the central region, whereas a small number are dispersed across other regions. To reduce the influence of population stratification on the GWAS results for each trait, we evaluated the effectiveness of GWAS correction using different numbers of principal components and ultimately included the first eight principal components and sex as covariates in the model. The genomic inflation factors for the GWAS analyses of the traits ranged from 0.97 to 1.02, indicating that the correction was effective.

### 3.3. Genetic Parameter Estimates

Table 2 presents the estimates of genetic parameters for BW and body size traits in Wenshang Barred chickens at the late rearing stage. Heritability estimates ranged from 0.18 ± 0.056 for CD to 0.48 ± 0.063 for KL, indicating low to moderate genetic contributions to phenotypic variation in these traits. Specifically, the heritability estimates for BW, CB, CD, KL, BOL, PW, SL, and SC were 0.44 ± 0.061, 0.38 ± 0.063, 0.18 ± 0.056, 0.48 ± 0.063, 0.37 ± 0.062, 0.39 ± 0.062, 0.32 ± 0.062, and 0.32 ± 0.063, respectively.

All estimated genetic correlations were positive and ranged from 0.61 to 0.98. The highest genetic correlation was observed between CB and CD (0.98 ± 0.14), whereas the lowest was observed between BOL and SC (0.61 ± 0.12). Likewise, all phenotypic correlations were positive, ranging from 0.14 to 0.77. The highest phenotypic correlation was observed between KL and BOL (0.77), whereas the lowest was observed between CD and SC (0.14).

### 3.4. Genome-Wide Association Study

**Figure 2:**
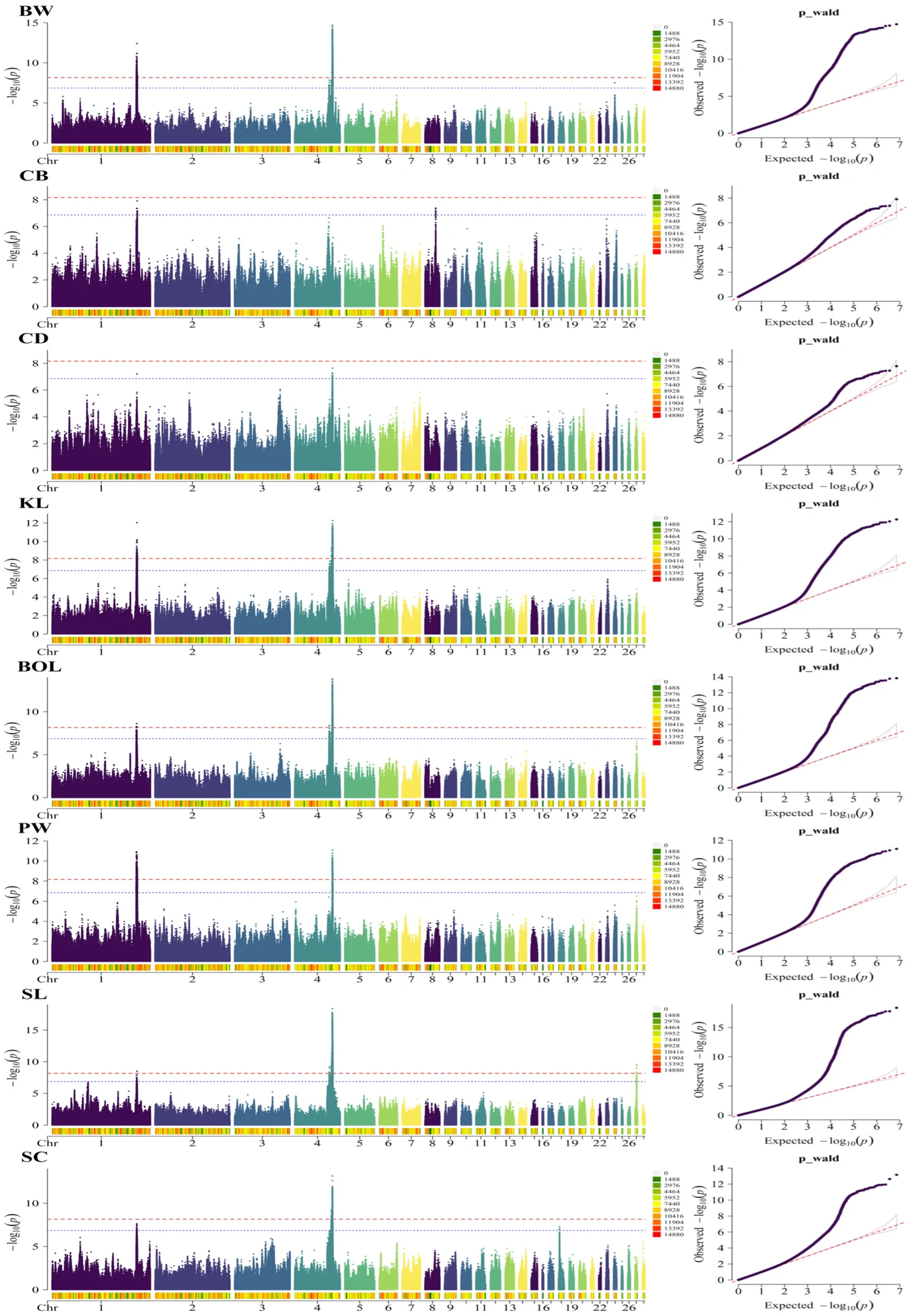
Manhattan and Q–Q plots of the genome-wide association study for BW and body size traits in Wenshang Barred chickens at the late rearing stage. Each dot represents a single SNP. The red and blue horizontal lines indicate the genome-wide significant threshold (*p* ≈ 6.91 × 10^−9^) and suggestive significant threshold (*p* ≈ 1.38 × 10^−7^), respectively.

Based on phenotypic data from 925 Wenshang Barred chickens and 7,235,426 quality-controlled SNPs, we identified 8211 significantly associated SNPs, of which 4080 reached the genome-wide significance threshold. These SNPs were mapped to 89 candidate genes (Supplementary Tables S2–S9). As shown in Figure 2, the Manhattan plots revealed distinct association peaks for all traits, with the major signals concentrated on chromosomes 1 and 4. Shared association regions across multiple traits were located at 168.03–170.24 Mb on chromosome 1, 68.24–70.22 Mb on chromosome 4, and 72.48–76.42 Mb on chromosome 4. The chromosome 1 region spanning 168.03–170.24 Mb was associated with BW, CB, CD, KL, BOL, PW, SL, and SC. The chromosome 4 region spanning 68.24–70.22 Mb was associated with BW, KL, BOL, and SL. The chromosome 4 region spanning 72.48–76.42 Mb was also significantly associated with multiple traits and contained numerous significant SNPs for BW, KL, BOL, PW, SL, and SC.

For individual traits, 2190 significant SNPs were identified for BW, primarily on chromosomes 1 and 4. A total of 23 and 18 significant SNPs were identified for CB and CD, respectively. For KL, BOL, and PW, 1599, 1209, and 1270 significant SNPs were identified, respectively, with the major association signals concentrated on chromosomes 1 and 4. For leg size traits, 1348 and 554 significant SNPs were identified for SL and SC, respectively. In addition to chromosomes 1 and 4, 27 significant SNPs for SL were detected in the 3.22–3.34 Mb region on chromosome 27, whereas three significant SNPs for SC were detected in the 2.22–2.28 Mb region on chromosome 18. Detailed information on the core association regions, numbers of significant SNPs, and candidate gene annotations is provided in Table 3, Table 4, Table 5 and Table 6. Genomic inflation factors ranged from 0.97 to 1.02 across traits, indicating no apparent systematic inflation in the GWAS results.

### 3.5. Regional Association, Linkage Disequilibrium, and Genotype–Phenotype Effect Analyses

**Figure 3:**
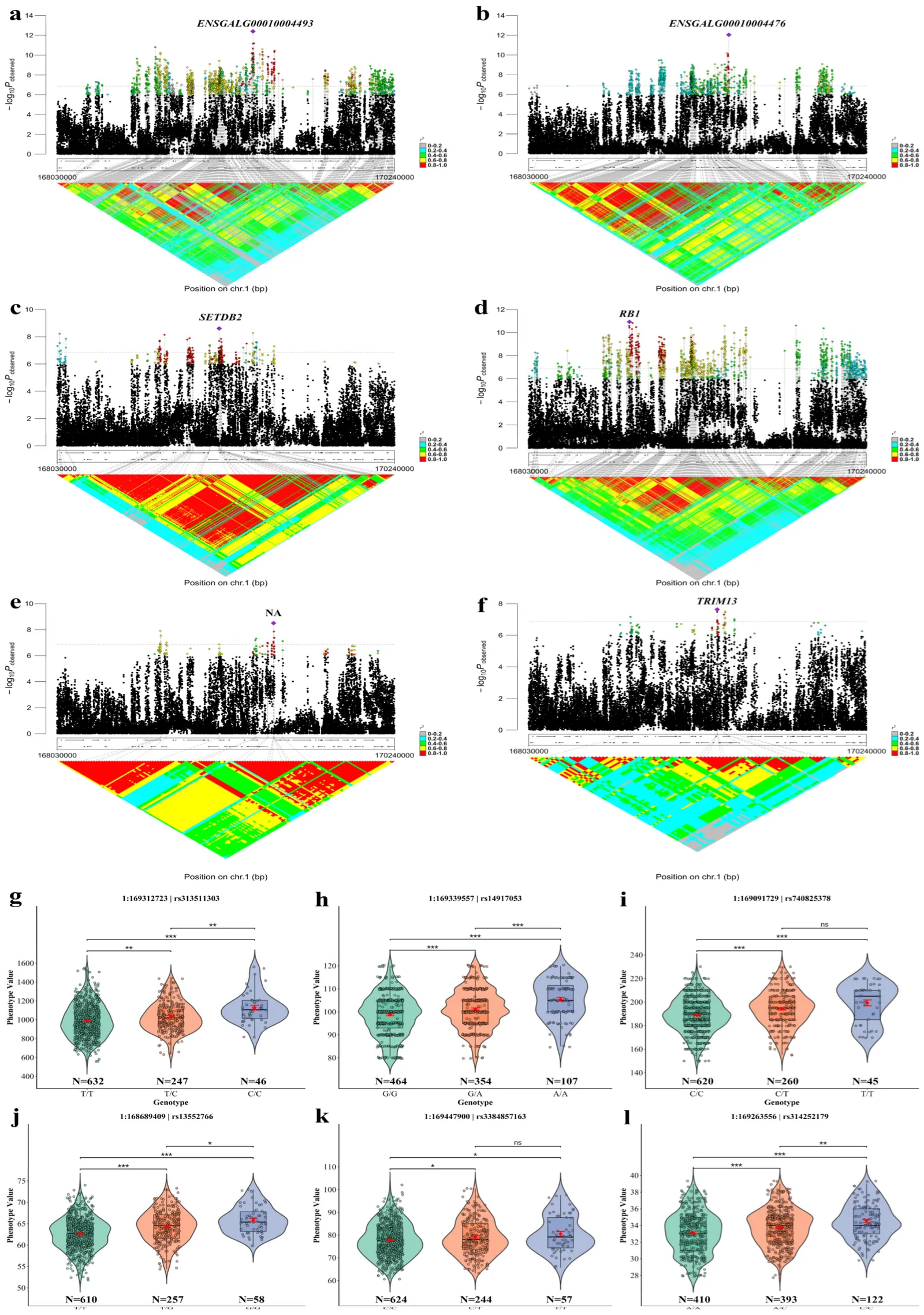
Regional association, linkage disequilibrium, and genotype–phenotype effect analyses for the GGA1:168.03–170.24 Mb region associated with BW and body size traits in Wenshang Barred chickens. Regional association and LD plots for BW, KL, BOL, PW, SL, and SC are shown in panels (**a**–**f**), respectively. Genotype–phenotype effect analyses of the lead SNPs for BW, KL, BOL, PW, SL, and SC are shown in panels (**g**–**l**), respectively. NA, the lead SNP was not annotated to any candidate gene. N, number of individuals with the indicated genotype. *, *p* < 0.05; **, *p* < 0.01; ***, *p* < 0.001; ns, not significant.

**Figure 4:**
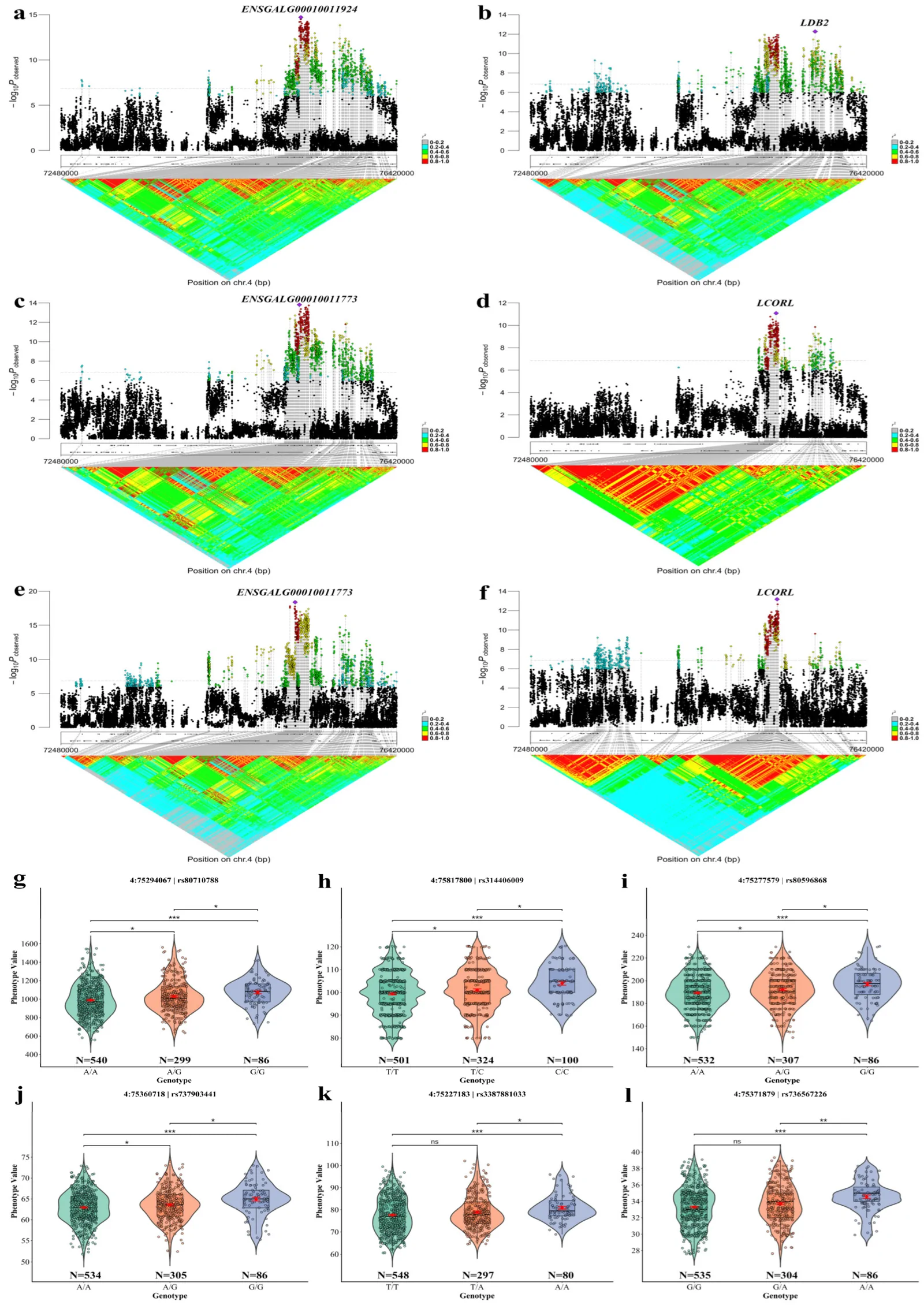
Regional association, linkage disequilibrium, and genotype–phenotype effect analyses for the GGA4:72.48–76.42 Mb region associated with BW and body size traits in Wenshang Barred chickens. Regional association and LD plots for BW, KL, BOL, PW, SL, and SC are shown in panels (**a**–**f**), respectively. Genotype–phenotype effect analyses of the lead SNPs for BW, KL, BOL, PW, SL, and SC are shown in panels (**g**–**l**), respectively. N, number of individuals with the indicated genotype. *, *p* < 0.05; **, *p* < 0.01; ***, *p* < 0.001; ns, not significant.

To further characterize the local genetic architecture of the major association signals and their genotype–phenotype relationships, regional association, linkage disequilibrium (LD), and lead-SNP genotype–phenotype effect analyses were performed for two shared significant association regions, GGA1:168.03–170.24 Mb and GGA4:72.48–76.42 Mb. Detailed information on the lead SNPs for the major association signals is presented in Table 7 and Figure 3 and Figure 4.

The GGA1:168.03–170.24 Mb region contained multiple candidate genes, including *RB1*, *KPNA3*, *CAB39L*, *INTS6*, *MLNR*, *NUDT15*, *FNDC3A*, *SERPINE3*, *SETDB2*, *TRIM13*, *WDFY2*, *DLEU7*, and *MED4*. This region was associated with BW, KL, BOL, PW, SL, and SC. Regional association and LD analyses for these traits are shown in Figure 3a–f. Most significant SNPs were clustered within regions of moderate-to-strong LD, and the lead SNP for each trait was located within a corresponding high-LD block. The genotype–phenotype effect analyses of the lead SNPs are shown in Figure 3g–l. For BW, KL, PW, and SC, phenotypic values increased significantly with the number of effect-allele copies. Notably, the BOL-associated SNP rs740825378 was located within an intron of *SETDB2* and upstream of *CAB39L*. The PW-associated SNP rs13552766 was located within an intron of *RB1*, whereas the SC-associated SNP rs314252179 was located downstream of *TRIM13*.

The GGA4:72.48–76.42 Mb region contained multiple candidate genes, including *KCNIP4*, *LAP3*, *LCORL*, *LDB2*, *NCAPG*, *PACRGL*, *PPARGC1A*, *PROM1*, *QDPR*, and *SLIT2*. This region was also significantly associated with BW, KL, BOL, PW, SL, and SC. Regional association and LD analyses are presented in Figure 4a–f. Most significant SNPs in this region showed moderate-to-strong LD, and the lead SNP for each trait was located within or adjacent to a corresponding high-LD block. As shown in Figure 4g–l, phenotypic values for BW, KL, BOL, and PW increased significantly with the number of effect-allele copies. The KL-associated SNP rs314406009 was located within an intron of *LDB2*. Meanwhile, the SNPs rs737903441 and rs736567226, associated with PW and SC, respectively, were both located within introns of *LCORL*.

### 3.6. Functional Enrichment Analysis

**Figure 5:**
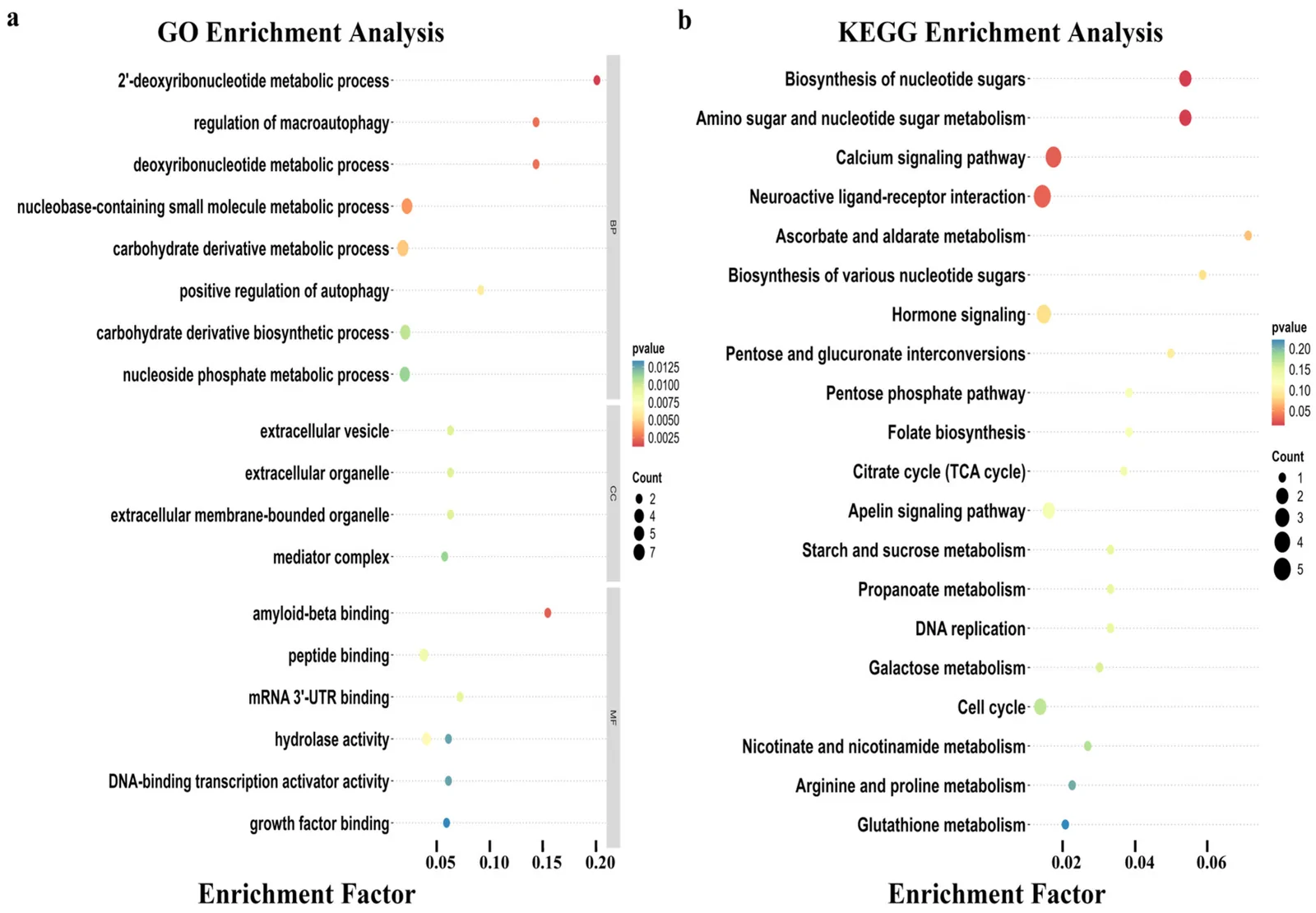
GO and KEGG enrichment analyses of candidate genes associated with body weight and body size traits in Wenshang Barred chickens. (**a**) The top 20 GO terms ranked by nominal *p* value; (**b**) the top 20 KEGG pathways ranked by nominal *p* value. Bubble size represents the number of candidate genes annotated to each term or pathway, and bubble color represents the nominal *p* value.

To characterize the functional profiles of the candidate genes, GO and KEGG enrichment analyses were performed for 89 candidate genes (Supplementary Tables S10 and S11). At a nominal threshold of *p* < 0.05, 41 GO terms and four KEGG pathways were nominally enriched (Figure 5).

Among the GO enrichment results, 22 terms were assigned to the biological process (BP) category. These terms were primarily related to nucleotide and carbohydrate-derivative metabolic processes, including 2′-deoxyribonucleotide metabolic process (GO:0009394) and deoxyribonucleotide metabolic process (GO:0009262). The cellular component (CC) category contained nine enriched terms, mainly involving extracellular organelle-related components, including extracellular organelle (GO:0043230) and extracellular membrane-bounded organelle (GO:0065010). Ten enriched terms were assigned to the molecular function (MF) category, primarily involving molecular binding and enzymatic activity, including amyloid-beta binding (GO:0001540) and hydrolase activity (GO:0016810).

KEGG enrichment analysis identified four nominally enriched pathways, including amino sugar and nucleotide sugar metabolism (gga00520), biosynthesis of nucleotide sugars (gga01250), calcium signaling pathway (gga04020), and neuroactive ligand–receptor interaction (gga04080). These four pathways are considered candidate pathways for BW and body size traits. Collectively, the candidate pathways were mainly related to nucleotide sugar metabolism and signal transduction. The top 20 GO terms and KEGG pathways ranked by nominal *p* value are displayed in Figure 5.

## 4. Discussion

BW and body size traits are important economic traits targeted in livestock and poultry breeding and are also considered indicators of animal health status. At 16 weeks of age, indigenous chickens are generally at the late rearing stage, during which growth is relatively slow and production costs may increase substantially. Strengthening selection for growth traits during this period may improve carcass and production performance, thereby enhancing economic efficiency. In the present study, approximately 10× whole-genome resequencing data from a mixed-sex population of 925 16-week-old Wenshang Barred chickens were used to estimate genetic parameters and perform GWAS, LD analysis of key association regions, genotype–phenotype effect analysis, and functional enrichment analysis for eight growth traits. We identified 4080 genome-wide significant SNPs, annotated 89 candidate genes, and identified four KEGG pathways at a nominal *p* < 0.05 threshold. These findings provide an important reference for understanding the genetic basis of growth traits in Wenshang Barred chickens at the late rearing stage.

Genetic parameters generally include heritability, genetic correlations, and phenotypic correlations. They can be used to reveal the extent to which phenotypic traits are influenced by genetic factors and the coordinated relationships among traits, and provide an important basis for evaluating genetic improvement potential, formulating breeding programs, and optimizing multi-trait selection strategies. However, relatively systematic reports on genetic parameter estimation at the late rearing stage are limited. In the present study, heritability estimates for BW and body size traits in Wenshang Barred chickens at the late rearing stage ranged from 0.18 to 0.48, indicating that these traits were influenced by genetic factors to a low-to-moderate extent. The heritabilities of BW and KL were 0.44 and 0.48, respectively, suggesting that they have relatively high genetic improvement potential. Therefore, BW and KL can serve as important candidate traits for the coordinated selection and breeding of growth traits at the late rearing stage. The heritabilities of CB and CD were 0.38 and 0.18, respectively; the heritabilities of BOL, KL, and PW ranged from 0.37 to 0.48; and the heritabilities of SL and SC were both 0.32. In the present study, phenotypic correlations among growth traits, including body weight and body size traits, ranged from 0.14 to 0.77. The phenotypic correlation between KL and BOL was the highest (0.77), whereas that between CD and SC was the lowest (0.14). Meanwhile, all genetic correlations among growth traits, including body weight and body size traits, were strong and positive, ranging from 0.61 to 0.98. The genetic correlation between CB and CD was the highest (0.98), whereas that between BOL and SC was the lowest (0.61). Genetic correlations among most traits were higher than the corresponding phenotypic correlations, suggesting that different body weight and body size traits may share a strong common genetic basis, whereas environmental effects may weaken their phenotypic associations.

These results are generally consistent with the overall trends of genetic parameter estimates for growth traits, including body weight and body size traits, in other chicken populations. For example, Liu et al. reported that the heritability estimates of growth traits, including body weight, metatarsal traits, and carcass traits, ranged from 0.07 to 0.61 and from 0.18 to 0.76 in white broilers and Guangxi yellow broilers, respectively. Liu et al. found that BW at 7 weeks of age in white broilers had positive genetic correlations of 0.31–0.84 with growth traits such as metatarsal length, metatarsal circumference, chest angle, and BOL. Abdominal fat weight also showed positive genetic correlations of 0.31–0.58 with growth traits such as BW, metatarsal length, KL, and BOL. In Guangxi yellow broilers, BW at 125 days of age had genetic correlations of 0.30–0.99 with other growth traits [24]. In four Egyptian dual-purpose chicken strains, the heritability estimates of growth traits, including body weight and body size traits, ranged from 0.24 to 0.52, and the heritabilities of BW16, SL, and KL were 0.30, 0.49, and 0.41, respectively. Across all combinations of body weight and body size traits, genetic correlations ranged from 0.47 to 0.64, whereas phenotypic correlations ranged from 0.25 to 0.41 [18]. In Ghanaian local chickens, the heritabilities of BW22 and multiple carcass traits ranged from 0.50 to 0.69. The Ghanaian local chicken study also showed that BW22 had moderate-to-high positive genetic and phenotypic correlations with most carcass traits [25]. Similarly, the heritability of BW at 16 weeks of age in Betong chickens was 0.47, which also indicates that body weight at the late rearing stage has a good genetic basis for selection. In Betong chickens, the genetic and phenotypic correlations between BW16 and breast meat weight were 0.63 and 0.68, respectively [17]. Therefore, selection for BW and body size traits at the late rearing stage may contribute to improvements in overall growth and some carcass traits. These results support the pattern of generally positive genetic associations between body weight and body size traits observed in the present study and suggest that selection for BW, KL, or other highly correlated body size traits may be accompanied by coordinated changes in related traits.

In the present study, the GGA4:72.48–76.42 Mb region was significantly associated with BW, KL, BOL, PW, SL, and SC in Wenshang Barred chickens at the late rearing stage. Previous studies have shown that this region is an important regulatory region for chicken growth and development [10,26,27]. In this region, we identified multiple candidate genes, including *LCORL*, *NCAPG*, *KCNIP4*, *PACRGL*, *SLIT2*, *QDPR*, *LDB2*, and *PROM1*. Among these genes, *LCORL*, *NCAPG*, *KCNIP4*, *SLIT2*, and *LDB2* have been repeatedly reported in studies of body weight or body size traits in livestock and poultry [28,29,30,31].

*NCAPG* encodes a subunit of the condensin I complex and plays a central role in chromosome condensation and cell division. It is a key component involved in the regulation of cell proliferation [32]. *LCORL* participates in the regulation of the growth hormone/insulin-like growth factor-1 (GH/IGF-1) signaling pathway, thereby affecting growth and development [33]. The *KCNIP4* gene belongs to the potassium channel-interacting protein family. Members of this family have broad physiological functions, including neurotransmitter release, smooth muscle contraction, heart rate regulation, and insulin secretion [28]. The *KCNIP4* gene participates in the negative-feedback regulation of the Wnt/β-catenin pathway [34]. The Wnt/β-catenin signaling pathway broadly regulates multiple biological processes, including stem-cell self-renewal, organ morphogenesis, and tumorigenesis [35,36]. *SLIT2* plays a key role in cell adhesion and migration [37]. *SLIT2* can downregulate the expression of Src tyrosine kinase in macrophages and inhibit the PI3K/AKT signaling pathway, thereby suppressing the differentiation of macrophages into osteoclasts and reducing bone resorption, thus affecting skeletal development [38]. *LDB2* encodes a LIM domain-binding protein. *LDB2* can affect cell proliferation, invasion, and migration by activating the p38 MAPK/ERK1/2/JNK signaling pathways and regulating the cellular immune microenvironment [39].

However, *PACRGL*, *QDPR*, and *PROM1* have been less frequently reported in studies of body size traits in livestock and poultry. Reports on the *PACRGL* gene are limited. Liu et al. (2023) showed that circPACRGL can promote cell proliferation, migration, and invasion and inhibit apoptosis by regulating the miR-330-3p/CNBP signaling axis [40]. The *QDPR* gene encodes quinoid dihydropteridine reductase and catalyzes the regeneration of tetrahydrobiopterin (BH4). BH4 is a cofactor in monoamine synthesis, phenylalanine hydroxylation, and nitric oxide production and participates in neurotransmitter biosynthesis [41,42]. *PROM1* plays a key role in photoreceptor development and the maintenance of photoreceptor homeostasis. Variants in this gene can cause inherited retinal dystrophies with diverse clinical manifestations and may subsequently affect animal feeding behavior [43].

In addition, upstream of the GGA4:72.48–76.42 Mb region, we identified an important association region, GGA4:68.24–70.22 Mb. Several candidate genes, including *TBC1D1*, *UGDH*, *KLF3*, *WDR19*, and *FAM114A1*, were identified in this region. The GGA4:68.24–70.22 Mb region was associated with BW, KL, BOL, and SL. This region is approximately 2.3 Mb away from the GGA4:72.48–76.42 Mb region and has been less explored in poultry breeding studies at the late rearing stage. Among these genes, *TBC1D1* negatively regulates muscle satellite-cell proliferation by mediating the cytoplasmic retention of the AS160–STAT3 complex [44]. *UGDH* can participate in the regulation of the PI3K/AKT signaling pathway [45], whereas *KLF3* can enhance cell viability by targeting *YBX1* and participating in the PI3K/AKT/mTOR signaling pathway [46].

In addition to the classical regulatory region for chicken growth and development on chromosome 4, we identified a region with an important association signal on chromosome 1, GGA1:168.03–170.24 Mb. Multiple candidate genes, including *RB1*, *KPNA3*, *CAB39L*, *INTS6*, *MLNR*, *NUDT15*, *FNDC3A*, *SERPINE3*, *SETDB2*, *TRIM13*, *WDFY2*, *DLEU7*, and *MED4*, were identified in this region. The GGA1:168.03–170.24 Mb region was associated with BW, CB, CD, KL, BOL, PW, SL, and SC. This region has been less explored in poultry breeding studies at the late rearing stage. In the present study, SNPs significantly associated with body size traits and BW within this region were densely distributed in moderate-to-strong LD blocks, suggesting that the GGA1:168.03–170.24 Mb region may contain loci associated with BW and body size traits in Wenshang Barred chickens at the late rearing stage. Among these loci, the most significant SNPs for BOL, PW, and SC were identified in *SETDB2* and *RB1*, and downstream of *TRIM13*, respectively.

Among these genes, *RB1* participates in the regulation of chicken preadipocyte proliferation. *RB1* can inhibit the transition of cells from the G1 phase to the S phase, thereby suppressing preadipocyte proliferation [47]. Upregulated expression of *CAB39L* can inhibit cell proliferation, reduce the expression levels of *PFKP* and *LDHA*, promote cell apoptosis, and enhance activation of the AMPK pathway [48]. *WDFY2* can activate insulin-induced Akt2 signaling by regulating the endosomal localization of the insulin receptor and IRS1/2 in hepatocytes [49]. Previous genome-wide association analyses have identified *MLNR* as a candidate gene associated with chicken growth. The *MLNR* gene is closely related to the digestive system, participates in the regulation of gastrointestinal motility and gastric emptying, and affects chicken growth and development [50]. *SETDB2* can inhibit cell proliferation both in vivo and in vitro and regulate cellular iron homeostasis through H3K9me3 modification [51]. In addition, *SETDB2* has been reported to participate in suppressing the transition of fibroblasts into myofibroblasts [52]. *KPNA3* specifically regulates the nuclear import and condensation of *RUNX2*, and knockout of this gene inhibits osteoblast differentiation [53]. *TRIM13* is an E3 ubiquitin ligase involved in maintaining central nervous system (CNS) homeostasis. *TRIM13* overexpression inhibits cell proliferation through an autophagy-mediated mechanism [54]. *INTS6* has been recognized as a potential tumor suppressor and can regulate the Wnt/β-catenin signaling pathway and epithelial–mesenchymal transition (EMT) [55]. *SERPINE3* can participate in protecting retinal pigment epithelial cells from oxidative stress damage, thereby potentially affecting the feeding behavior of chickens [56].

To further elucidate the biological functions of the candidate genes, enrichment analysis was conducted for the identified candidate genes. A total of four candidate pathways (*p* < 0.05) with potential genetic regulatory relationships with body weight and body size traits in Wenshang Barred chickens at the late rearing stage were identified: amino sugar and nucleotide sugar metabolism (gga00520), biosynthesis of nucleotide sugars (gga01250), calcium signaling pathway (gga04020), and neuroactive ligand–receptor interaction (gga04080). Among these pathways, gga00520 and gga01250 have been less frequently reported in studies of poultry growth traits. These two pathways are key pathways involved in amino sugar and nucleotide sugar metabolism and may affect body weight and body size traits by regulating glycosylation modification, intracellular and extracellular matrix synthesis, cell proliferation, and tissue development. Ca^2+^ signaling is closely associated with skeletal muscle development, homeostasis, and regeneration. The gga04020 pathway participates in the regulation of skeletal muscle development, homeostasis, and regeneration, as well as osteoblast differentiation and bone remodeling [57,58]. The gga04080 pathway participates in the regulation of information transmission between neural signals and peripheral tissues. The regulation of feed intake during animal development is crucial for maintaining growth performance and meat production in livestock and poultry. In chickens, feeding behavior is jointly controlled by central neural and peripheral regulation and is coordinated by the nervous and digestive systems [59]. Previous studies have shown that neuroactive ligands play key roles in the regulation of appetite in chickens [60] and are associated with growth rate and egg-laying performance [61].

Several limitations need to be considered with respect to the results of this study. This GWAS was conducted in only one population of Wenshang Barred chickens at 16 weeks of age, and this study design cannot determine whether these findings are consistent at different ages or in other populations. The associated loci and genetic markers have not been replicated in other independent populations; therefore, their robustness and suitability for use in selection remain uncertain. Candidate genes were identified primarily on the basis of their overlap with associated SNPs or their location near these SNPs (within 5 kb); although this identification approach is commonly used in previous GWAS studies, this study did not conduct gene expression analyses or experimental validation of molecular mechanisms, and the available data cannot determine which genes underlie the association signals or establish that they have a causal effect on the traits studied. In addition, GO and KEGG enrichment analyses used a nominal significance threshold of *p* < 0.05 without correction for multiple testing; therefore, the resulting pathways should be regarded as exploratory leads rather than mechanistic evidence. These limitations do not negate the associations and exploratory findings observed in this study population, but they constrain causal interpretation and claims of immediate use in selection; future studies need to replicate the findings in other independent populations and conduct targeted expression and functional studies of prioritized candidate genes to strengthen biological interpretation and the assessment of breeding applications.

## 5. Conclusions

Using whole-genome resequencing data, we performed GWAS of body weight and body size traits in 925 Wenshang Barred chickens at the late rearing stage. We identified 4080 genome-wide significant SNPs and annotated 89 candidate genes and four candidate pathways. We identified the classical regulatory region for growth traits on GGA4:72.48–76.42 Mb, which contains candidate genes including *NCAPG*, *LCORL*, *KCNIP4*, *SLIT2*, *LDB2*, *PACRGL*, *QDPR*, and *PROM1*. In addition, an important association region, GGA4:68.24–70.22 Mb, was identified approximately 2.3 Mb upstream of this region, in which *TBC1D1*, *UGDH*, and *KLF3* may serve as candidate genes for body weight and body size traits. Furthermore, we identified an important association region on chromosome 1, GGA1:168.03–170.24 Mb, in which *RB1*, *CAB39L*, *WDFY2*, *MLNR*, *SETDB2*, *KPNA3*, *TRIM13*, *INTS6*, and *SERPINE3* may serve as candidate genes for body weight and body size traits. Enrichment analysis suggested that amino sugar and nucleotide sugar metabolism, biosynthesis of nucleotide sugars, the calcium signaling pathway, and neuroactive ligand–receptor interaction may serve as candidate pathways for body weight and body size traits. These findings clarify the genetic basis of body weight and body size traits in Wenshang Barred chickens at the late rearing stage and may provide a basis for further evaluation of genetic markers in indigenous chicken breeding.

## Figures and Tables

**Table 1:** Descriptive statistics of body weight and body size traits in Wenshang Barred chickens at the late rearing stage (16 weeks of age).

Trait	N	Mean	SD	Min	Max	CV (%)
BW (g)	925	1009.89	176.98	558.00	1561.00	17.52
CB (mm)	925	50.56	3.81	39.60	62.02	7.53
CD (mm)	925	77.68	11.07	50.82	110.00	14.25
KL (mm)	925	100.47	8.99	80.00	120.00	8.94
BOL (mm)	925	190.98	15.45	150.00	230.00	8.09
PW (mm)	925	63.33	3.89	51.82	74.01	6.14
SL (mm)	925	78.35	7.77	60.59	102.07	9.92
SC (mm)	925	33.56	2.38	28.00	39.00	7.10

Note: N, number of individuals; SD, standard deviation; CV, coefficient of variation; BW, body weight; CB, chest breadth; CD, chest depth; KL, keel length; BOL, body oblique length; PW, pelvic width; SL, shank length; SC, shank circumference. All body size traits were expressed in millimeters (mm).

**Table 2:** Estimates of genetic parameters for BW and body size traits in Wenshang Barred chickens at the late rearing stage.

Trait	BW	CB	CD	KL	BOL	PW	SL	SC
BW	**0.44** **(0.061)**	0.92 (0.06)	0.87 (0.13)	0.78 (0.07)	0.89 (0.06)	0.97 (0.05)	0.84 (0.08)	0.92 (0.06)
CB	0.52 (0.03)	**0.38** **(0.063)**	0.98 (0.14)	0.67 (0.10)	0.80 (0.09)	0.89 (0.06)	0.73 (0.10)	0.87 (0.08)
CD	0.26 (0.03)	0.26 (0.03)	**0.18** **(0.056)**	0.94 (0.07)	0.97 (0.07)	0.86 (0.13)	0.74 (0.10)	0.78 (0.18)
KL	0.46 (0.03)	0.38 (0.03)	0.56 (0.02)	**0.48** **(0.063)**	0.91 (0.03)	0.73 (0.08)	0.73 (0.08)	0.63 (0.11)
BOL	0.50 (0.03)	0.39 (0.03)	0.57 (0.02)	0.77 (0.02)	**0.37** **(0.062)**	0.90 (0.08)	0.74 (0.08)	0.61 (0.12)
PW	0.58 (0.02)	0.57 (0.02)	0.36 (0.03)	0.45 (0.03)	0.48 (0.03)	**0.39** **(0.062)**	0.77 (0.08)	0.80 (0.09)
SL	0.38 (0.03)	0.36 (0.03)	0.61 (0.02)	0.54 (0.03)	0.54 (0.03)	0.47 (0.03)	**0.32** **(0.062)**	0.72 (0.11)
SC	0.52 (0.03)	0.43 (0.03)	0.14 (0.03)	0.31 (0.03)	0.28 (0.03)	0.46 (0.03)	0.36 (0.03)	**0.32** **(0.063)**

Note: Heritability estimates are shown on the diagonal, genetic correlations above the diagonal, and phenotypic correlations below the diagonal. Standard errors are shown in parentheses.

**Table 3:** Summary of significantly associated SNPs for body weight traits in Wenshang Barred chickens at the late rearing stage.

Trait	Chr	Base-Pair Region		N	Gene Annotation
		Start	End		
BW	1	168231159	170230264	997	*ARL11*/*ATP7B*/*CAB39L*/*CDADC1*/*CYSLTR2*/*DHRS12*/*DLEU7*/*FAM124A*/*FNDC3A*/*HTR2A*/*INTS6*/*ITM2B*/*KCNRG*/*KPNA3*/*LOC107051704*/*LOC112531567*/*LPAR6*/*MED4*/*MLNR*/*NUDT15*/*PHF11*/*RB1*/*RCBTB1*/*RCBTB2*/*RNASEH2B*/*SERPINE3*/*SETDB2*/*SPRYD7*/*SUCLA2*/*TMEM272*/*TRIM13*/*WDFY2*/*gga-mir-15a*/*gga-mir-16-1*
BW	4	68244622	69022235	9	*APBB2*/*FAM114A1*/*KLHL5*/*PDS5A*/*SMIM14*/*WDR19*
BW	4	72722294	76417871	1183	*C1QTNF7*/*CC2D2A*/*CCKAR*/*CD38*/*CLRN2*/*CPEB2*/*FBXL5*/*FGFBP1*/*FGFBP2*/*KCNIP4*/*LAP3*/*LCORL*/*LDB2*/*LOC121110525*/*MED28*/*NCAPG*/*PACRGL*/*PPARGC1A*/*PROM1*/*QDPR*/*RBPJ*/*TAPT1*
BW	24	2559010		1	*JAM3*

Note: BW, body weight; Chr, chromosome; Base-Pair Region, physical position of the associated genomic region; N, number of significantly associated SNPs within the corresponding base-pair region.

**Table 4:** Summary of significantly associated SNPs for chest size traits in Wenshang Barred chickens at the late rearing stage.

Trait	Chr	Base-Pair Region		N	Gene Annotation
		Start	End		
CB	1	169091729	170131593	15	*CAB39L*/*DHRS12*/*RNASEH2B*/*SETDB2*/*WDFY2*
CB	8	21320783	21424529	8	*DMBX1*/*VDHAP*
CD	1	169452007		1	
CD	4	75166597	75378175	17	*LCORL*/*LOC121110525*

Note: CB, chest breadth; CD, chest depth; Chr, chromosome; Base-Pair Region, physical position of the associated genomic region; N, number of significantly associated SNPs within the corresponding base-pair region.

**Table 5:** Summary of significantly associated SNPs for trunk size traits in Wenshang Barred chickens at the late rearing stage.

Trait	Chr	Base-Pair Region		N	Gene Annotation
		Start	End		
KL	1	168085125	170097794	580	*ARL11*/*CAB39L*/*CDADC1*/*DLEU7*/*FAM124A*/*FNDC3A*/*INTS6*/*KCNRG*/*KPNA3*/*LOC107051704*/*LOC112531567*/*LRCH1*/*MLNR*/*PHF11*/*RB1*/*RCBTB1*/*RCBTB2*/*RNASEH2B*/*SERPINE3*/*SETDB2*/*SPRYD7*/*SUCLA2*/*TRIM13*/*WDFY2*/*gga-mir-15a*/*gga-mir-16-1*
KL	4	68777481	70212140	25	*C4ORF19*/*DTHD1*/*SMIM14*/*UGDH*
KL	4	72711388	76353237	994	*CC2D2A*/*CCDC149*/*CD38*/*CLRN2*/*DHX15*/*FGFBP1*/*FGFBP2*/*KCNIP4*/*LAP3*/*LCORL*/*LDB2*/*LOC121110525*/*MED28*/*NCAPG*/*PACRGL*/*PPARGC1A*/*PROM1*/*QDPR*/*TAPT1*/*TBC1D19*
BOL	1	168033116	169452007	85	*CAB39L*/*CDADC1*/*FNDC3A*/*LPAR6*/*LRCH1*/*PHF11*/*RB1*/*RCBTB2*/*SETDB2*/*SPRYD7*/*TRIM13*
BOL	4	68759039	70212140	57	*C4ORF19*/*KLF3*/*KLHL5*/*PGM2*/*RELL1*/*SMIM14*/*TBC1D1*/*WDR19*
BOL	4	72722294	76142828	1067	*CCKAR*/*CD38*/*CLRN2*/*DHX15*/*FGFBP1*/*FGFBP2*/*KCNIP4*/*LAP3*/*LCORL*/*LDB2*/*LOC121110525*/*MED28*/*NCAPG*/*PACRGL*/*PROM1*/*QDPR*/*TAPT1*
PW	1	168066389	170234473	918	*ARL11*/*ATP7B*/*CAB39L*/*CDADC1*/*DHRS12*/*DLEU7*/*FAM124A*/*FNDC3A*/*HTR2A*/*INTS6*/*ITM2B*/*KPNA3*/*LOC107051704*/*LOC112531567*/*LPAR6*/*LRCH1*/*MED4*/*MLNR*/*NUDT15*/*PHF11*/*RB1*/*RCBTB1*/*RCBTB2*/*RNASEH2B*/*SERPINE3*/*SETDB2*/*SPRYD7*/*SUCLA2*/*TMEM272*/*TRIM13*/*WDFY2*
PW	4	75153979	76030080	352	*CLRN2*/*LAP3*/*LCORL*/*LDB2*/*LOC121110525*/*NCAPG*/*QDPR*/*TAPT1*

Note: KL, keel length; BOL, body oblique length; PW, pelvic width; Chr, chromosome; Base-Pair Region, physical position of the associated genomic region; N, number of significantly associated SNPs within the corresponding base-pair region.

**Table 6:** Summary of significantly associated SNPs for leg size traits in Wenshang Barred chickens at the late rearing stage.

Trait	Chr	Base-Pair Region		N	Gene Annotation
		Start	End		
SL	1	168705854	169507240	17	*CAB39L*/*RB1*/*RCBTB2*/*SETDB2*
SL	4	68738655	69683315	54	*C4ORF19*/*DTHD1*/*FAM114A1*/*RELL1*/*SMIM14*/*TBC1D1*/*UGDH*
SL	4	72708942	76415245	1250	*C1QTNF7*/*CC2D2A*/*CD38*/*CLRN2*/*CPEB2*/*DHX15*/*FGFBP1*/*FGFBP2*/*KCNIP4*/*LAP3*/*LCORL*/*LDB2*/*LOC121110521*/*LOC121110525*/*MED28*/*NCAPG*/*PACRGL*/*PPARGC1A*/*PROM1*/*QDPR*/*SLIT2*/*TAPT1*/*TBC1D19*
SL	27	3226456	3330987	27	*ABI3*/*ATP5G1*/*CALCOCO2*/*GNGT2*/*IGF2BP1*/*PHOSPHO1*/*SNF8*/*UBE2Z*/*ZNF652*
SC	1	168695971	169376093	12	*KCNRG*/*RB1*/*TRIM13*
SC	4	72489111	76123829	539	*CCDC149*/*DHX15*/*FGFBP1*/*FGFBP2*/*KCNIP4*/*LCORL*/*LDB2*/*LGI2*/*LOC121110521*/*LOC121110525*/*NCAPG*/*PACRGL*/*PPARGC1A*/*PROM1*/*SLC34A2*
SC	18	2225198	2270734	3	

Note: SL, shank length; SC, shank circumference; Chr, chromosome; Base-Pair Region, physical position of the associated genomic region; N, number of significantly associated SNPs within the corresponding base-pair region.

**Table 7:** Allele information and association statistics of lead SNPs for major association signals of body weight and body size traits in Wenshang Barred chickens.

Trait	Chr	Ps	SNP	Allele	MAF	*β* ± se	*p*
BW	1	169312723	rs313511303	T/C	0.18	67.21 ± 9.13	4.00 × 10^−13^
BW	4	75294067	rs80710788	A/G	0.26	62.65 ± 7.75	1.92 × 10^−15^
KL	1	169339557	rs14917053	G/A	0.31	3.38 ± 0.47	9.03 × 10^−13^
KL	4	75817800	rs314406009	T/C	0.28	3.51 ± 0.48	5.43 × 10^−13^
BOL	1	169091729	rs740825378	C/T	0.19	5.42 ± 0.90	2.48 × 10^−9^
BOL	4	75277579	rs80596868	A/G	0.26	6.03 ± 0.77	1.52 × 10^−14^
PW	1	168689409	rs13552766	T/G	0.20	1.54 ± 0.22	1.19 × 10^−11^
PW	4	75360718	rs737903441	A/G	0.26	1.41 ± 0.20	8.28 × 10^−12^
SL	1	169447900	rs3384857163	C/T	0.19	2.04 ± 0.34	3.16 × 10^−9^
SL	4	75227183	rs3387881033	T/A	0.25	2.76 ± 0.30	4.17 × 10^−19^
SC	1	169263556	rs314252179	A/C	0.34	0.59 ± 0.11	2.33 × 10^−8^
SC	4	75371879	rs736567226	G/A	0.26	0.83 ± 0.11	6.83 × 10^−14^

Note: Chr, chromosome; Ps, physical position (bp); SNP, single-nucleotide polymorphism; Allele, the two alleles reported for the SNP; MAF, minor allele frequency; *β* ± se, estimated SNP effect ± standard error; *P*, association *p* value.

## Data Availability

The whole-genome resequencing data generated in this study have been deposited in the Genome Sequence Archive (GSA) at the National Genomics Data Center, China National Center for Bioinformation/Beijing Institute of Genomics, Chinese Academy of Sciences (CNCB-NGDC; https://ngdc.cncb.ac.cn/gsa/), under accession numbers CRA049271 and CRA049265. The datasets will be publicly available upon publication of this article.

## Supplementary Materials

The following supporting information can be downloaded at: https://www.mdpi.com/article/10.3390/ani16203255/s1, Table S1. Overview of whole-genome resequencing data for the experimental samples. Tables S2–S9. Significantly associated SNPs for body weight and body size traits and their gene annotations. Tables S10 and S11. Results of Gene Ontology and Kyoto Encyclopedia of Genes and Genomes enrichment analyses.

## Abbreviations

The following abbreviations are used in this manuscript:

SNP	Single-nucleotide polymorphism
GGA	Gallus gallus chromosome
LD	Linkage disequilibrium
BW	Body weight
CB	Chest breadth
CD	Chest depth
KL	Keel length
BOL	Body oblique length
PW	Pelvic width
SL	Shank length
SC	Shank circumference
